# Genetic Variation in miR-146a Is Not Associated with Susceptibility to IgA Nephropathy in Adults from a Chinese Han Population

**DOI:** 10.1371/journal.pone.0139554

**Published:** 2015-10-01

**Authors:** Bin Yang, Wei Wei, Yunying Shi, Zhuochun Huang, Bei Cai, Junlong Zhang, Binwu Ying, Lanlan Wang

**Affiliations:** 1 Department of Laboratory Medicine, West China Hospital, Sichuan University, 610041 Chengdu, China; 2 West China School of Medicine, Sichuan University, 610041 Chengdu, China; 3 Department of Nephrology, West China Hospital, Sichuan University, 610041 Chengdu, China; Universitat Pompeu Fabra, SPAIN

## Abstract

**Background:**

MicroRNA 146a (miR-146a) is a 19 to 23 nucleotide long, small non-coding RNA with gene regulatory functions that has influence on the pathogenesis of many diseases. A single nucleotide polymorphism (rs2910164 C>G) in pre-miR-146a is correlated with the expression of miR-146a. The aim of this study was to perform an association analysis of rs2910164 with IgA nephropathy in adult patients from a Chinese Han population.

**Methods:**

A total of 145 patients with renal biopsy-proved IgA nephropathy (IgAN) and 179 healthy controls were recruited to the current study. rs2910164 was genotyped by the polymerase chain reaction (PCR) and high-resolution melting methods (HRM). Clinical characteristics and pathology grading of patients with IgAN were recorded at the time of kidney biopsy.

**Result:**

There were significant differences among the population of patients grouped by different age of onset in a co-dominant model (CG *vs*. CC *vs*. GG) (*p* = 0.033) and a recessive model (CG+CC *vs*. GG) (*p* = 0.001). However, no significant difference was observed in the distribution of genotypes between cases and controls (*p* = 0.144). There was also no significant difference between rs2910164 and patient quantitative traits (all *p* > 0.003) or different pathology grading (Lee’s grading system and tubular atrophy/interstitial fibrosis in the Oxford classification) (all *p* > 0.05).

**Conclusions:**

There was no association of rs2910164 with susceptibility to IgAN in adults from a Chinese Han population. However, rs2910164 was correlated with the age of onset of IgAN in adult patients.

## Introduction

Primary IgA nephropathy (IgAN) is the most common chronic glomerular disease worldwide [1]. Clinical manifestations of IgAN are usually macroscopic hematuria, hypertension, and different degrees of proteinuria [2]. The disease is characterized by deposition of pathogenic polymeric immunoglobulin A1 (IgA1) and presentation of complement, especially C3 [1, 3–5]. The IgA1 that deposits in the mesangium can lead to an inflammatory cascade that injures the kidneys [1, 6]. About 25% to 50% of patients will progress to end-stage renal disease (ESRD) within 20 years [7, 8]. Moreover, individuals of Pacific Asian origin have a higher risk of progression to ESRD than those of European origin [9]. Although research on IgAN has been ongoing for decades, the molecular and genetic mechanisms are still not fully understood, and a disease-specific treatment is lacking.

MicroRNAs (miRNAs) are a group of 19 to 23 nucleotide long, non-coding RNAs that function as post-transcriptional regulators of targeted mRNAs by binding to the 3’-untranslated region (3’-UTR) and causing the degradation or translation repression of mRNAs, thereby inhibiting gene expression [10, 11]. Recent studies have revealed important roles of miRNAs in regulating components in mammalian toll-like receptors (TLRs) signaling and innate immunity pathways [12–14]. There is currently evidence suggesting that aberrant expression of miRNAs is associated with several immune diseases, such as Graves’ ophthalmopathy, rheumatoid arthritis, and IgAN [15–17].

miR-146a is well known for its important role in regulation of immune and inflammatory response [18]. Its target proteins, interleukin-1 receptor-associated kinase 1 (IRAK1) and TNF receptor associated factor 6 (TRAF6), are important components in TLRs and pro-inflammatory signaling pathways [10, 19, 20]. Inhibition of IRAK1 can decrease expression of interferon γ (IFN-γ), which is a typical helper T (Th) 1 cytokine, and lead to abnormal IgA1 glycosylation in IgAN by influencing production of Th2 cytokines [21–23]. TRAF6 is associated with NF-кB activation, which can influence synthesis of tumor necrosis factor α (TNF-α). TNF-α can increase interleukin-6 (IL-6) production, leading to inflammatory responses in the glomerular mesangium through a series of steps in IgAN [24–26]. Recent studies have revealed that the increased expression of miR-146a occurs in patients with IgAN [27, 28], suggesting that miR-146a may be significantly associated with IgAN.

Single nucleotide polymorphisms (SNPs), a type of genetic variation that can contribute to variation of human phenotype, can regulate expression of miRNAs, which in turn affects some aspects of disease, such as individual susceptibility [29]. In miR-146a, the functional SNP rs2910164 C>G is located in the pre-miR-146a of miR-146a. Previous studies showed that rs2910164 could affect the expression level of mature-miR-146a [30–32], but those studies did not mention which one of the mature forms of miR-146a (hsa-miR-146a-3p or hsa-miR-146a-5p) were studied. Those studies demonstrated that rs2910164 was associated with many diseases, including severe sepsis [30], systemic lupus erythematosus [33], and chronic periodontitis [34], all of which have an inflammatory background. However, the relationship between rs2910164 and IgAN has only been reported in one study. That study revealed that a higher frequency of rs2910164 C allele was associated with higher morbidity of childhood IgAN [35].

As noted previously, individuals of Pacific Asian origin have a higher risk of progression to ESRD than those of European origin. The frequencies of rs2910164 C allele and G allele distributed very differently in pacific Asian individuals and European individuals. We found that the frequency of C allele: frequency of G allele of rs2910164 in Chinese individuals is 0.646: 0.354, that in Japanese individuals is 0.610: 0.390, and that in European individuals is 0.325: 0.675 (ss52076591, Genotype Freq, http://www.ncbi.nlm.nih.gov/projects/SNP/snp_ref.cgi?rs=2910164). Pacific Asian individuals clearly have a higher frequency of rs2910164 C allele than European individuals. This led us to hypothesize that rs2910164 C allele might be associated with the etiology and progression of IgAN. In this study, therefore, we investigated whether the rs2910164 is associated with IgAN in adults from a Chinese Han population.

## Materials and Methods

### Study subjects

This study was approved by the Institutional Review Board of Sichuan University. All participants provided their written informed consent to participate in this study. A total of 145 patients who had renal biopsy-proved IgAN in West China Hospital of Sichuan University, and 179 gender-, age-, and ethnicity-matched healthy controls were enrolled in this study. The mean ages of patients and controls were 33.5 ± 9.7 (range 18 to 58) years and 35.5 ± 10.2 (range 15 to 58) years, respectively (*p* = 0.915). Males accounted for 48% (69/145) of patients and females for 52% (76/145). Amongst controls, 55% (99/179) were male and 45% (80/179) were female. All study subjects were of Han ethnicity and lived in the West of China. Every case was an adult with diagnosed IgAN. Demographic data are shown in Table 1.

**Table 1:** Demographic, clinical, and histological data in controls and IgAN patients.

Parameter	Control	IgAN	*p*-value
Number of subjects	179	145	
Age (years)	35.50 ± 10.23 (15–58)	33.53 ± 9.66 (18–58)	0.915
Gender (M:F)	0.55:0.45	0.48:0.52	0.167
Male	99 (55%)	69 (48%)	
Female	80 (45%)	76 (52%)	
SBP (mmHg)	119 ± 14 (89–160)	121 ± 15 (91–175)	0.741
DBP (mmHg)	65 ± 12 (52–100)	76 ± 11 (50–115)	0.325
Hemoglobin (g/dL)	149 ± 17 (103–210)	134 ± 21 (96–182)	0
Platelets (10^9^/L)	207 ± 55 (166–327)	175 ± 60 (78–349)	0
Proteinuria (g/24 h)	-	1.34 (0.69–2.78)	-
sAlb (g/L)	48.3 ± 2.1 (23.9–89.2)	39.7 ± 10.1 (18.6–77.3)	0
Scr (μmol/L)	80.2 ± 14.9 (68.1–127.8)	102.4 ± 58.1 (40.8–377.1)	0.001
BUN (mmol/L)	4.55 ± 1.18 (1.37–8.41)	6.50 ± 3.26 (2.59–19.42)	0
eGFR (mL/min/1.73 m^2^)	90 ± 14 (59–162)	83 ± 34 (14–224)	0.06
sIgA (g/L)	2.12 ± 0.13 (1.6–2.6)	2.73 ± 1.25 (0.40–6.78)	0.157
C3 (g/L)	0.90 ± 0.17 (0.24–1.41)	0.93 ± 0.25 (0.13–1.56)	0.336
C4 (g/L)	0.21 ± 0.08 (0.11–0.37)	0.26 ± 0.14 (0.10–0.87)	0.008
Lee’s grading system			
Grade I	-	12 (8.27%)	
Grade II	-	34 (23.45%)	
Grade III	-	59 (40.69%)	
Grade IV- V	-	40 (27.59%)	
The Oxford classification of tubular atrophy/interstitial fibrosis			
0–25%	-	130 (89.66%)	
26–50%	-	15 (10.34%)	
> 50%	-	0	

Data are mean ± SD or median interquartile range, and comparisons between groups were made by the Kruskal-Wallis test as appropriate. WBC, white blood cell; SBP, systolic blood pressure; DBP, diastolic blood pressure; BUN, blood urea nitrogen; sIgA, serum immunoglobulin A; C3, complement 3; C4, complement 4; Scr, serum creatinine; sAlb, serum albumin; eGFR, estimated glomerular filtration rate.

IgAN was determined by a renal biopsy, demonstrating a dominant IgA deposition in the mesangium on immunofluorescence microscopy. Clinical characteristics of patients with IgAN were recorded at the same time of kidney biopsy, including age of onset, the course of disease, clinical symptoms, tonsillitis, pharyngitis, systolic/diastolic blood pressure, hemoglobin, platelet count, albumin, serum creatinine (Scr), blood urea nitrogen (BUN), 24-h urinary protein, levels of immunoglobulin A (IgA), C3, and C4, and estimated glomerular filtration rate (eGFR) (using a modification of the modified diet in renal disease equation based on the Chinese chronic kidney disease (CKD) population [36]). Pathology grading was recorded for each individual at the same time. Data are shown in Tables 1 and 2.

**Table 2:** Genotype and allele distributions of rs2910164 C>G.

Parameter	Controls (n = 179)	IgAN patients (n = 145)	*p*-value	OR (95% CI)
HWE*	0.086	0.501		
Genotype				
CC	74 (41.34%)	59 (40.69%)		1
GC	90 (50.28%)	64 (44.14%)	0.144	0.892 (0.598–1.425)
GG	15 (8.38%)	22 (15.17%)	NS	1.206 (0.584–2.489)
Alleles				
G	120 (33.52%)	108 (37.24%)	0.324	1
C	238 (66.48%)	182 (62.76%)	NS	0.850 (0.615–1.175)

Comparisons between groups were made by the χ2 test as appropriate.

*P-value of Hardy-Weinberg equilibrium; NS: no significance.

### Genomic DNA extraction

Blood samples (3 mL) were collected in EDTA-coated tubes, and genomic DNA was isolated from whole blood samples using the whole blood DNA kit (Roche Diagnostics; Penzberg, Bavaria, Germany). DNA was extracted from 200 μL of the whole blood, according to the manufacturer’s protocol. The extracted DNA was assessed for purity, yield, and concentration on a spectrophotometer (Bio-RAD; Hercules, CA, USA). Purity was monitored by the A260/A280 ratio. DNA was diluted to 10 ng/μL for working solutions, and isolated DNA was stored at -20°C.

### Polymerase chain reaction and high-resolution melting method

The rs2910164 polymorphism in the pre-miR-146a was assessed. The polymerase chain reaction (PCR) and melting curve analyses were performed under the same conditions in a 96-well plate on the Light Cycler 480 (Roche Diagnostics). The primers were designed to a small fragment surrounding the polymorphisms and avoided the presence of other sequence variations in the primer region. rs2910164 PCR primers were 5’-CATGGGTTGTGTCAGTGTCAGAGCT-3’ (forward) and 5’-TGCCTTCTGTCTCCAGTCTTCCAA-3’ (reverse). Reaction mixtures contained 1.0 μL purified genomic DNA (10 ng/μL), 0.5 μL forward primer, 0.5 μL reverse primer, 1.4 μL 20×EVA-GREEN, 0.5 μL dNTP (10 mM), 0.2 μL Hot Star Taq® Plus DNA polymerase, 2 μL 10× buffer, and 1 μL 50 mM MgCl_2_. Real-time PCR was performed with the following conditions: an initial denaturation step at 95°C for 15 min, continued with 50 cycles of 95°C for 10 s, 60°C for 15 s, and 72°C for 20 s. After the amplification phase, a melting curve analysis was performed at 95°C for 1 min, 40°C for 1 min, 65°C for 1 s, and finally slow heating at 0.01°C/s to 95°C.

### HRM analysis

Collected data were analyzed by the Light Cycler 480 Gene Scanning software v1.2 (Roche Diagnostics). Software programs used the following analysis method: normalization by selecting linear regions before and after the melting transition, temperature shifting by selecting the threshold, then automatic grouping by calculation. The exact same setting of the normalization was used for all experiments. The genotype of each subset was defined, according to known genotypes of controls.

### Statistical analysis

The Hardy-Weinberg equilibrium (HWE) for rs2910164 was tested by the χ^2^ test with df = 1. The difference of the frequency distribution was analyzed by the χ^2^ test. A difference of *p* < 0.05 was considered statistically significant. The difference in data for clinical features and pathology grading among different genotypes was analyzed using the Kruskal—Wallis tests. For testing of multiple factors, the Bonferroni method was used to correct the significant levels and *p* < 0.003 was considered statistically significant. All analyses above were performed with SPSS software (version 19.0, SPSS; Chicago, IL, USA). Statistical power analysis was done with Quanto software (version 1.2.4; University of Southern California; Los Angeles, CA, USA) to determine the power to detect the association of rs2910164 alleles with susceptibility to IgAN at the designated significance level [37–39].

## Results

### Determination of rs2910164 genotypes

**Fig 1:**
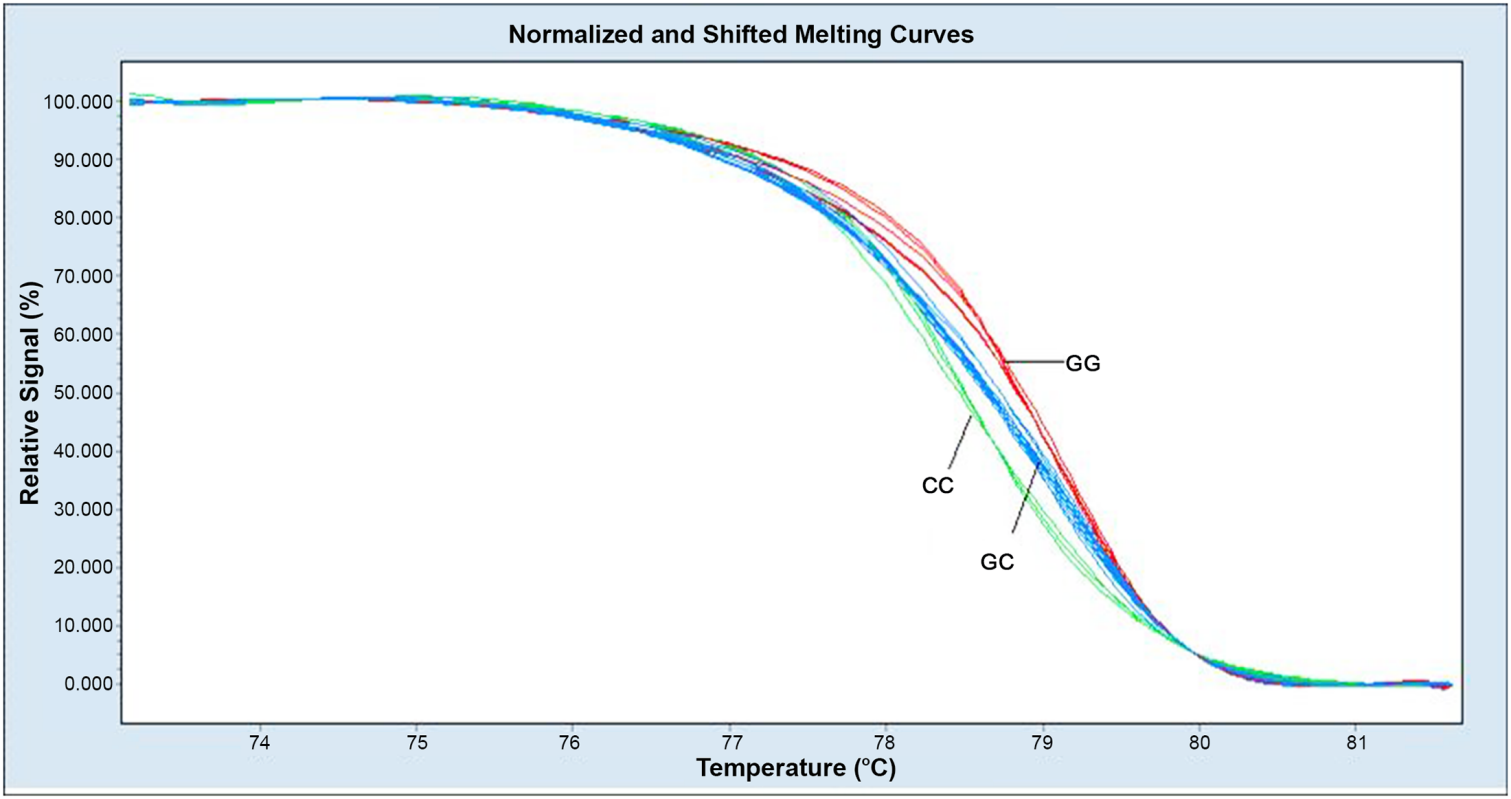
Melting profiles for the three genotypes from the normalized melting curves.

**Fig 2:**
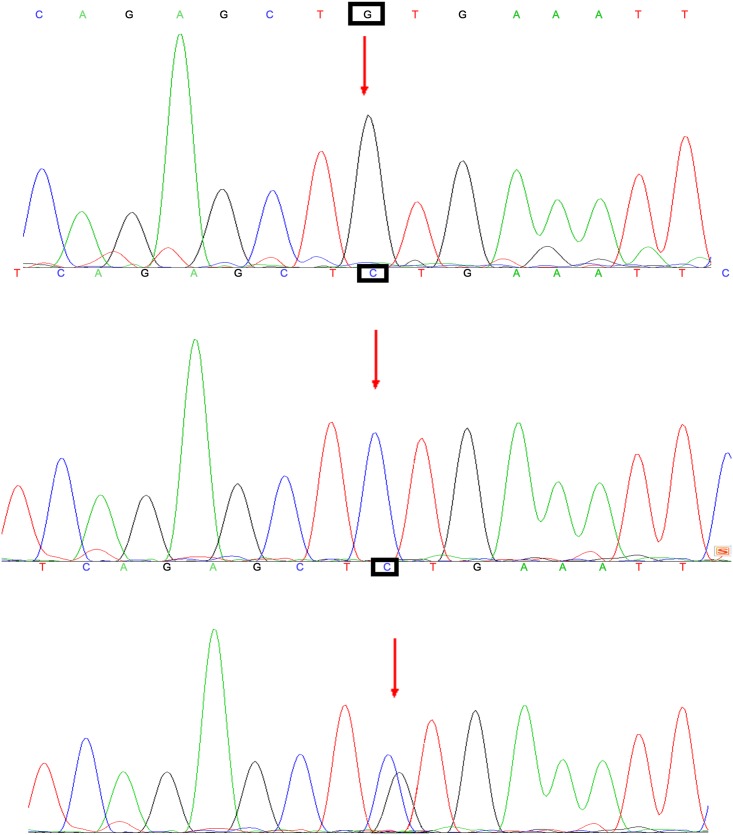
Representative sequencing results of rs2910164.

Three genotype melting profiles were clearly distinguished from the normalized melting curves. Difference plots are shown in Fig 1. Additional PCR products were randomly selected from the three genotype melting profiles. Representative sequencing results for rs2910164 are also shown in Fig 2.

### Association of rs2910164 genotypes and alleles with susceptibility to IgAN

Table 2 shows the genotype and allele frequencies of cases and controls. No significant deviation of genotypes was found either in cases or controls with Hardy-Weinberg equilibrium (both *p* > 0.05). There was no significant difference in the distribution of genotypes between IgAN patients and controls (*p* = 0.144). Frequencies of rs2910164 C allele and G allele between cases and controls were also analyzed, and no significant difference was observed (*p* = 0.324). This analysis had low power to detect an association (10% power at the suggested significance level for a minor allele with a frequency of 0.372 and OR = 0.892 in co-dominant model).

### Association between rs2910164 genotypes and clinical indexes of IgAN patients

IgAN patients were classified into three groups according to the three genotypes, CC, GC and GG, and the means of patient quantitative traits were calculated, including age of onset, white blood cell (WBC), neutrophil, and lymphocyte counts, systolic and diastolic blood pressure (SBP and DBP), blood urea nitrogen (BUN), serum immunoglobulin A (sIgA), Complement 3 and 4 (C3 and C4) levels, serum creatinine (Scr), serum albumin (sAlb), platelet counts, estimated glomerular filtration rate (eGFR) and proteinuria at 24 h. All the clinical indexes did not show significant differences (all *p* > 0.003). The proportion of patients in three age groups (18–30, 30–40 and over 40 years old) were then analyzed and the *p*-values revealed significant differences in both the co-dominant model (CG *vs*. CC *vs*. GG) (*p* = 0.033) and recessive model (CG+CC *vs*. GG) (*p* = 0.001). Data are shown in Tables 3 and 4.

**Table 3:** miR-146a SNP (rs1946518) genotypes and clinical indexes in IgAN patients.

Parameter	rs2910164			*p*-value
	CC	GC	GG	
Age of onset (years)	30 ± 9 (20–58)	32 ± 10 (18–50)	36 ± 9 (21–58)	0.038
WBC (10^9^/L)	7.17 ± 2.54 (4.67–14.69)	7.13 ± 2.94 (1.03–16.1)	7.88 ± 2.52 (4.31–12.2)	0.962
Neutrophils (10^9^/L)	63.3 ± 8.5 (48.9–83.2)	85.0 ± 9.32 (48.4–92.2)	65.0 ± 11.3 (41.9–81.7)	0.312
Lymphocytes (10^9^/L)	36.9 ± 48.0 (11.8–325.4)	25.5 ± 8.7 (3.7–46.3)	25.4 ± 11.9 (1.41–46.2)	0.319
SBP (mmHg)	120 ± 13 (91–140)	124 ± 15 (100–175)	122 ± 14 (100–155)	0.67
DBP (mmHg)	74 ± 13 (50–90)	78 ± 13 (60–115)	78 ± 10 (58–100)	0.32
BUN (mmol/L)	5.87 ± 2.25 (2.63–15.52)	6.99 ± 4.23 (2.59–19.42)	6.23 ± 2.51 (3.2–12.2)	0.751
sIgA (g/L)	2.81 ± 0.93 (0.91–6.29)	2.88 ± 1.46 (0.40–6.78)	2.36 ± 0.98 (0.46–3.86)	0.587
C3 (g/L)	0.88 ± 0.30 (0.24–1.56)	0.87 ± 0.24 (0.56–1.21)	0.90 ± 0.29 (0.13–1.12)	0.648
C4 (g/L)	0.25 ± 0.16 (0.13–0.87)	0.24 ± 0.13 (0.10–0.72)	0.24 ± 0.07 (0.14–0.38)	0.182
Scr (μmol/L)	91.2 ± 52.4 (40.8–377.1)	115.1 ± 67.1 (53.0–335.4)	94.36 ± 42.01 (60.0–216.0)	0.219
sAlb (g/L)	38.6 ± 7.0 (18.6–47.7)	39.1 ± 10.0 (18.6–74.4)	44.2 ± 17.3 (26.2–77.3)	0.58
Platelets (10^9^/L)	174 ± 55 (83–305)	172 ± 68 (78–349)	183 ± 53 (121–264)	0.907
eGFR (mL/min/1.73 m^2^)	90 ± 35 (18–224)	75 ± 35 (14–178)	80 ± 25 (23–111)	0.25
Proteinuria (g/24 h)	2.49 ± 3.21 (0.5–2.78)	2.31 ± 2.60 (0.59–2.33)	1.11 ± 1.24 (0–2.41)	0.241

Data are mean ± SD or median interquartile range, and comparisons between groups were made by the Kruskal-Wallis test as appropriate. WBC, white blood cell; SBP, systolic blood pressure; DBP, diastolic blood pressure; BUN, blood urea nitrogen; sIgA, serum immunoglobulin A; C3, complement 3; C4, complement 4; Scr, serum creatinine; sAlb, serum albumin; eGFR, estimated glomerular filtration rate.

**Table 4:** The proportion of IgAN patients who were grouped as co-dominant model and recessive model for different age groups.

Age of onset (years)	CC (n = 59)	GC (n = 64)	GG (n = 22)	*p*-value
18–30	35 (49.29%)	29 (40.85%)	7 (9.86%)	
30–40	17 (36.96%)	23 (41.07%)	6 (13.04%)	0.033
40 –	7 (25.00%)	12 (42.86%)	9 (32.14%)	
	CC/GC (n = 123)	GG (n = 22)		
18–30	64 (90.14%)	7 (9.86%)		
30–40	40 (86.96%)	6 (13.04%)		0.001
40 –	19 (67.86%)	9 (32.14%)		

Comparisons between groups were made by the χ2 test as appropriate.

### Association between rs2910164 genotypes and pathology grading of IgAN patients

The glomerular changes of IgAN patients were classified into grades I–V according to Lee’s grading system [40]. To evaluate glomerular changes, the light microscopy features of the renal biopsy were examined. Distribution of the genotype frequencies were then compared among different grades in subgroups classified by different illness times. We only analyzed one subgroup (illness time < 1 year) because the other subgroups had insufficient data. No significant difference was observed (*p* = 0.294). Because the extent of tubular atrophy/interstitial fibrosis in IgAN can better evaluate disease severity, IgAN patients were classified into three groups according to the Oxford classification [41]. The genotype frequency distributions were also compared among different grades in the same subgroup; however, there was no significant difference (*p* = 0.937). Data are shown in Table 5.

**Table 5:** Distribution of the genotype frequencies among different grades in subgroup (sick time < 1 year).

Parameter	CC (n = 33)	CG (n = 37)	GG (n = 12)	*p*-value
Lee's grading system				
Grade I	3 (9.09%)	2 (5.41%)	1 (8.33%)	
Grade II	10 (30.30%)	5 (13.51%)	3 (25.00%)	0.156
Grade III	14 (42.42%)	15 (40.54%)	7 (58.33%)	NS
Grade IV	5 (15.15%)	12 (32.43%)	0 (0%)	
Grade V	1 (3.03%)	3 (8.11%)	1 (8.33%)	
The Oxford classification of tubular atrophy/interstitial fibrosis				
0–25%	31 (93.94%)	34 (91.89%)	11 (91.67%)	0.936
26–50%	2 (6.06%)	3 (8.11%)	1 (8.33%)	NS

Comparisons between groups were made by the χ2 test as appropriate. NS: no significance.

## Discussion

In this case-control study of IgAN among adults from a Chinese Han population, we found an association between the onset age and rs2910164 in the recessive model and co-dominant model (*p* = 0.001 and 0.033, respectively). However, no significant difference in the distribution of genotypes was observed in rs2910164, which locates on the pre-miR-146a, between the IgAN patients and the controls (*p* = 0.358). We could not observe any association between the frequency of rs2910164 and susceptibility to IgAN in adult patients from a Chinese Han population. Hence, rs2910164 was also not associated with patient quantitative traits (Tables 1 and 3).

As noted previously, miR-146a, which is a specific form of miRNA, plays important roles in immune and inflammatory responses. It can influence its target proteins IRAK1 and TRAF6 and affect IFN-γ and TNF-α to influence susceptibility to IgAN or the disease course of IgAN. rs2910164 was demonstrated to influence the expression level of miR-146a [42]. Thus, rs2910164 may correlate with both susceptibility to and progression of IgAN.

In this study we did not observe any association of rs2910164 with susceptibility to IgAN, but the relatively low power of the study is a limitation. However, the difference between the age of onset in the recessive model and co-dominant model are both significant, suggesting that rs2910164 may play an important role in development of the disease.

We considered the reason why we did not find an association between rs2910164 and susceptibility to IgAN. Excluding the low power caused by small sample size, one possibility is that miR-146a increased as a consequence of IgAN rather than changing prior to disease development. As there was an association between rs2910164 and age of onset in IgAN, the aberrant expression of miR-146a caused by rs2910164 may regulate disease pathogenesis. As noted previously, miR-146a can decrease the inflammatory response. Previous studies showed that the rs2910164 C allele could cause mispairing within the hairpin and decrease the expression of miR-146a [43, 44]. Inflammatory response would be more active at lower levels of miR-146a than at higher levels. Active inflammatory responses could facilitate IgAN pathogenesis, which could explain why adults carrying the rs2910164 C allele had a younger age of disease onset than those with the rs2910164 G allele.

Moreover, recent studies also revealed that the rs2910164 C allele could lead to a higher morbidity of childhood IgAN [35]. This study result was similar to that of the current study because both studies found that young people who carried the rs2910164 C allele had a higher morbidity due to IgAN. Most researchers agree that human immunity is more active in younger populations compared to older populations. More active immunity could lead to more active inflammatory responses. miR-146a could depress the inflammatory responses, and the rs2910164 C allele could lead to a lower level of miR-146a than the rs2910164 G allele. Individuals with the C allele should have more active immune responses than individuals with the G allele. From the above, we conclude that young patients carrying the C allele would show higher morbidity of IgAN because they have more active inflammatory conditions. However, in an older population, immunity may be low and the inflammatory response may be impaired.

In this study we only found an association between age of onset of IgAN with rs2910164 (*p* = 0.038). The significant difference in the recessive and co-dominant models suggested that the rs2910164 C allele could promote the development of IgAN. However, a limitation was that we had too few study subjects, which may lead to an inaccurate result. A study with a larger sample size is needed in the future for conclusive results. Studies that record additional clinical aspects of subject data, such as serum levels of TNFα and IRAK1, are needed to improve our understanding of the molecular mechanism by which miR-146a might influence the development of IgAN.

In summary, in this study we did not observe any association between rs2910164 and susceptibility to IgAN in adults from a Chinese Han population. Moreover, the significant difference of age of onset suggested that rs2910164 might play a role in the development of IgAN in adult patients from a Chinese Han population. However, further analysis must be performed with larger numbers of samples to enhance the power of the study and expand the knowledge of the functions of the miR-146a polymorphism on the development of IgAN. Research to understand the relationship between miRNA expression patterns and physiological functions are just beginning. Through studies of SNPs of miRNAs we can improve our understanding of the potential influence of these SNPs on disease [45].

## Supporting Information

S1 Table Clinical and SNP data for patients and controls. (XLS)
